# High‐Resolution Self‐Assembly of Functional Materials and Microscale Devices via Selective Plasma Induced Surface Energy Programming

**DOI:** 10.1002/smll.202408822

**Published:** 2024-12-29

**Authors:** Luke J. Tinsley, Prakash Karipoth, James H. Chandler, Silvia Taccola, Pietro Valdastri, Russell A. Harris

**Affiliations:** ^1^ Future Manufacturing Processes Research Group, School of Mechanical Engineering University of Leeds LS2 9JT United Kingdom; ^2^ STORM Lab, School of Electrical Engineering University of Leeds LS2 9JT United Kingdom

**Keywords:** Atmospheric Plasma, current keywords: Self‐assembly, Flexible Electronics, Functional Materials, Microfabrication, Soft Robotics

## Abstract

Current technologies preclude effective and efficient self‐assembly of heterogeneous arrangements of functional materials between 10^−1^ and 10^−5^ m. Consequently, their fabrication is dominated by methods of direct material manipulation, which struggle to meet the designers’ demands regarding resolution, material freedom, production time, and cost. A two‐step, computer‐controlled is presented, multi‐material self‐assembly technique that allows heterogenous patterns of several centimeters with features down to 12.5 µm in size. First, a micro plasma jet selectively programs the surface energy of a polydimethylsiloxane substrate through localized chemical functionalization. Second, polar fluids containing functional materials are simplistically introduced which then self‐assemble according to the patterned regions of high surface energy over timescales of the order of seconds. In‐process control enables both high‐resolution patterning and high throughput. This approach is demonstrated to produce heterogenous patterns of materials with varying conductive, magnetic, and mechanical properties. These include magneto‐mechanical films and flexible electronic devices with unprecedented processing times and economy for high‐resolution patterns. This self‐assembly approach can disrupt the current lithography/direct write paradigm that dominates micro/meso‐fabrication, enabling the next generation of devices across a broad range of fields via a flexible, industrially scalable, and environmentally friendly manufacturing route.

## Graphical abstract

Self‐assembly via surface energy programming offers an environmentally friendly, efficient, and flexible micromanufacturing approach for functional devices. The process uses a selective atmospheric plasma discharge to induce super‐hydrophilicity and subsequently control the patterning of inks containing functional materials. Significant benefits versus current state of the art are demonstrated through the fabrication and characterization of a heater, sensor, and actuator.

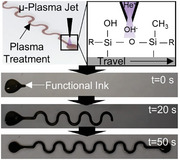

## 1 Introduction

**Figure 1:**
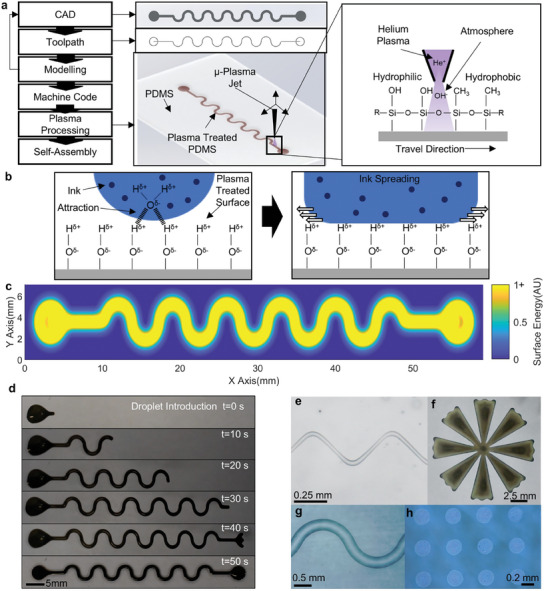
An overview of SA by surface energy programming. a) Process flow for SA via surface energy patterning with the CAD, toolpath and plasma processing steps detailed. b) The mechanism for ink self‐assembly. c) Modelling of the surface energy increase resulting from plasma treatment for a given parameter set and toolpath. d) Time lapse of SA of serpent pattern once a droplet is introduced. e–h) Demonstrations of self‐assembled patterns: e) 30 µm zig zag with PEDOT:PSS ink. f) Large flower with silver ink. g) Serpentine pattern with PEDOT:PSS ink. h) Array of 0.25 mm dots with cobalt ferrite ink.

The structured arrangement of functional materials in the dimensional range of 10^−5^ m to 10^−1^ m is critical to realizing functionality in many devices and applications. Biological systems employ self‐assembly (SA) across these dimensions to realize hierarchical multi‐material architectures of unparalleled complexity. Emulating this approach has been recognized as a route to revolutionizing the manufacturing of synthetic devices.^[^ ^1^, ^2^, ^3^, ^4^, ^5^ ^]^ SA has been leveraged with great success in nano and micro‐scale fabrication where direct manipulation of the molecular or atomic scale building blocks is challenging, time‐consuming, and expensive.^[^ ^1^ ^]^ Several mechanisms exist to drive this behavior, such as base pairing with DNA,^[^ ^6^ ^]^ chemical interactions,^[^ ^7^ ^]^ or molecular interactions,^[^ ^8^ ^]^ to highlight some. However, these mechanisms scale poorly for the creation of structures in the range of 10^−5^ m to 10^−1^ m due to the small initial size of the constituent elements and growing influence of gravitational forces, making fabrication slow, costly, and prone to defects.^[^ ^9^ ^]^ SA in this dimensional range is typically part‐to‐part, where sub‐assemblies combine or shape‐morph through a mechanism such as magnetic alignment.^[^ ^10^, ^11^ ^]^ This makes these approaches incapable of realizing continuously heterogeneous structures, and requires a separate fabrication protocol for the initial sub‐assemblies. More recently, large scale SA spanning 7 cm was demonstrated for micro/nano‐particle monolayers whereby particles suspended in ethanol spread across a water surface driven by surface tension gradients.^[^ ^12^ ^]^ However, this process is not capable of heterogenous patterning, with the self‐assembly shape dictated by the container, and is limited to monolayers. Direct manipulation of matter is possible in some instances at these scales, with technologies such as ink jet printing,^[^ ^13^ ^]^ aerosol jet printing,^[^ ^14^ ^]^ electrohydrodynamic printing,^[^ ^15^ ^]^ or lithographic techniques^[^ ^16^ ^]^ which can pattern micro‐features using materials with a wide range of mechanical, electrical, magnetic, and chemical properties. While lithographic techniques can fabricate structures with high resolution, the process is time‐consuming, expensive, requires specialist infrastructure, and uses environmentally harmful chemicals.^[^ ^16^ ^]^ Furthermore, it restricts the possible patterns and their flexibility of design changes due the template‐based nature. On the contrary, the other processes listed leverage digital control, where CAD data is used to drive selective processing. This provides benefits, including: device personalization at no extra fabrication cost; easy and rapid design iteration; and accessible techniques for non‐experts from a plethora of fields. However, each of these processes bring limitations on materials, resolution, and typically has, for parts spanning several centimeters and consisting of small features, long unit processing times.^[^ ^17^, ^18^, ^19^, ^20^, ^21^, ^22^, ^23^ ^]^ In addition, these processes are associated with low throughput, poor scalability, and high unit cost, rendering them uneconomical for many applications, whilst acting as a barrier to innovation in the new devices they could create. Here we present an alternative to the template‐based and selective processing archetypes which overcomes their respective limitations, whereby SA is driven through the forces arising due to spatial programming of the substrate's surface energy with a bespoke computer‐controlled micro plasma jet (**Figure** **1**a–d). This represents a breakthrough from previous works in this area by demonstrating new orders of capability in resolution, process control, materials, and optimization enabled by novel apparatus.^[^ ^24^, ^25^ ^]^ The increased surface energy results from plasma‐substrate interactions whereby non‐polar methyl functional groups, native to the polydimethylsiloxane (PDMS) substrate, are replaced with highly polar silanol groups (Figure 1a).^[^ ^26^ ^]^ While this change is specific to silicones, similar mechanisms of plasma induced hydrophilicity have been demonstrated for many polymers.^[^ ^27^, ^28^ ^]^ Due to the localization of the plasma discharge, the change in chemical composition of the surface occurs selectively, resulting in gradients of polar surface energy that can be defined to control the flow of polar fluids (Figure 1b–d, Video S1, Supporting Information). Furthermore, using a localized atmospheric plasma discharge removes the need for the templates/masks associated with selective processing using global plasma treatment, or the additional infrastructure and process steps related to commonly deployed low‐pressure global plasma treatment methodologies. This approach was used to demonstrate the highly controlled SA of representative functional inks containing; silver nanoparticles, poly(3,4‐ethylenedioxythiophene):poly(styrene sulfonate) (PEDOT:PSS), and hard magnetic cobalt ferrite nanoparticles (CoFe_2_O_4_) (Figure 1e–h, formulation in Table S2, Supporting Information). Via modulation of the plasma ignition conditions, it was possible to vary the propagation of active species to control the resolution of SA continuously between 12.5 µm and 3 mm with a system latency of 20 ms. This ability re‐defines the relationship between resolution and processing time typically associated with selective processing techniques as the resolution can be varied in‐process according to specific part requirements to minimize processing time. Furthermore, the continuously variable resolution enables accurate fabrication where features are not limited to, for example, dimensions in discrete multiples of the nozzle size. Finally, compared to current techniques, this approach is also environmentally friendly due to the use of water as the primary solvent, near‐zero material waste, small amounts of ink required, and low power consumption. This unique combination of characteristics position the technique as a high resolution, accurate, flexible, sustainable, and economically scalable manufacturing solution to deliver impact in both research and industrial contexts.

**Figure 2:**
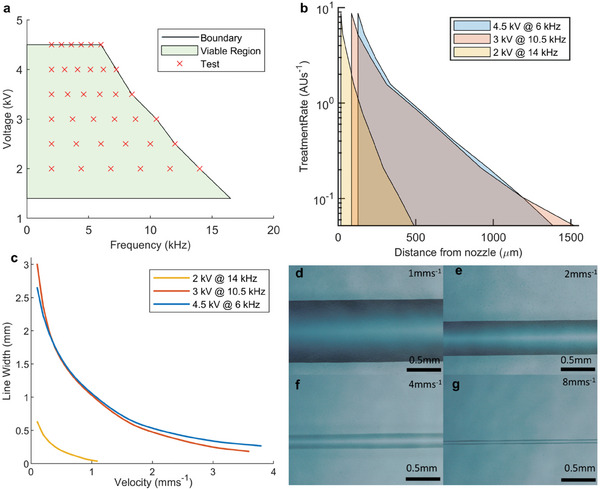
Characterization of the effects of applied voltage, frequency and speed on process resolution. a) Viable region for plasma ignition parameters and combinations selected for characterization b) Distribution of surface energy increase rate resulting from selected plasma ignition parameters. c) Predicted line width vs speed for selected parameter combinations. d–g) Line produced when operating using 4.5 kV at 6 kHz and PEDOT:PSS ink at d) 1 mms^−1^ e) 2 mms^−1^ f) 4 mms^−1^ g) 8 mms^−1^.

**Figure 3:**
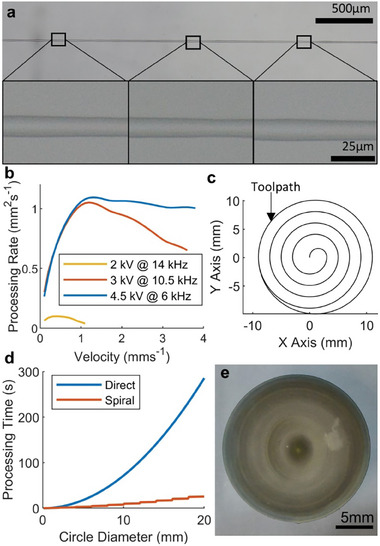
Process optimization for speed and resolution. a) 12.5 µm line with PEDOT:PSS ink b) Area treatment rate vs velocity for selected parameters with PEDOT:PSS ink using direct treatment approach. c) Example toolpath for spiral infill approach. d) Comparison of processing times using direct and concentric processing approaches. e) Self‐assembled 20 mm diameter circle using silver ink.

## 2 Characterization and Optimization

Compared to other selective processing techniques, this approach uniquely separates material deposition from its positioning, which are typically simultaneous and co‐dependent. This introduces the opportunity to independently change the characteristics of each, greatly extending overall capabilities and eliminating compromising relationships, such as that between nozzle size and material throughput in deposition‐based processes. These benefits are then compounded by control over the spatial distribution of treatment rate with low latency through modulation of the plasma ignition parameters to enable real‐time resolution control. While previous research has identified a large range of parameters that influence the behavior of the plasma discharge,^[^ ^29^, ^30^, ^31^ ^]^ those which would provide real‐time dynamic control with short response times were selected here. Therefore, the influence of applied voltage, frequency, and scan speed on the plasma behavior and subsequent SA were explored. These were investigated to find a viable region for continuous operation of the process without interruption, thereby avoiding variability and extended processing times (**Figure** **2**a). At voltages above 4.5 kV, nozzle breakages became frequent, while below 4.5 kV the frequency was limited by the maximum current of the high voltage amplifier. While active species were still produced below 1.4 kV, the weaker discharge resulted in long processing times so was not considered viable. Images of the plasma discharge at selected parameter combinations can be seen in Figure S1 (Supporting Information). Using the identified viable parameter region, 36 voltage‐frequency combinations were selected to investigate to give in‐depth understanding of their relationship. Through mapping of the stationary discharge characteristics, the SA pattern could be predicted from a given scan speed, toolpath, and parameter set. This was accomplished by characterizing the relationship between the treatment rate and radial distance from the nozzle, then subsequently calculating the spatial distribution of the plasma dose based on any given scan speed and toolpath (Figure 2b,c). This approach predicted the line width with an average error of <5% (σ = 4%) compared to actual lines (method in Supporting information S3, Figure S2, Supporting Information). This rapid characterization technique, requiring only 40 s of processing time, 20 µl of ink, and 324 mm^2^ of substrate per parameter combination will facilitate the development of a diverse ink and substrate library in the future. Furthermore, regular calibration using the technique can ensure process stability through variable atmospheric conditions such as pressure and humidity, and through system evolution such as the nozzle wear resulting from the plasma discharge (≈0.25 µm per hour, see methods).

These experiments, which used a nozzle diameter of 8.7 µm, revealed a non‐linear relationship between the voltage and frequency, and the resulting dose and distribution (Figure 2b; Figures S3,S4, Supporting Information for full dataset). The computed line width versus velocity relationships for selected parameter sets, chosen to highlight key trends in the process characteristics, can be seen in Figure 2c, with Figure 2d–g showing images of lines produced using 4.5 kV/6 kHz at speeds from 1–8 mms^−1^. Considering the three parameter sets shown in Figure 2b, the total treatment rate (the rate at which the surface energy increases) increases substantially between 2 kV/14 kHz‐3 kV/10.5 kHz, with only marginal increases from 3 kV/10.5 kHz‐4.5 kV/6 kHz. However, there is a significant difference in the spatial distribution of treatment between 3 kV/10.5 kHz‐4.5 kV/6 kHz. At 125 µm from the nozzle, the treatment rate for 4.5 kV/6 kHz is 65% greater than that of 3 kV/10.5 kHz, with the rates reaching equivalence at 1185 µm, after which 3 kV/10.5 kHz had a higher treatment rate. This contradicts previous reports that found increasing the voltage leads to a greater spread of plasma.^[^ ^29^ ^]^ This difference was attributed to the interdependence of the gas flow rate with applied voltage/frequency, which are inversely proportional (Figure S5, Supporting Information). At 2 kV/14 kHz, the treatment rate is substantially lower at all distances, but the higher spatial focus enables smaller lines when the speed is compensated appropriately. Therefore, the selection of optimal parameters is highly dependent on the specificities of the desired design. Here two common optimization scenarios are considered: speed and resolution. To optimize for speed, the system was operated at 4.5 kV/6 kHz, as this corresponded to the highest treatment rates at short distances from the nozzle, while increasing the speed until the dose was too low to induce SA. Using this approach, the minimum mean continuous line width achieved was 65 µm (59–71 µm) at a speed of 8 mms^−1^ (Figure 2g). Operating beyond this speed resulted in non‐continuous SA. To optimize for resolution, a fixed speed of 0.2 mms^−1^ and frequency of 15 kHz were used while decreasing the voltage until the SA line was no longer continuous. This resulted in a minimum mean line width of 12.5 µm (10.56–14.08 µm) corresponding to a voltage of 1.4 kV (**Figure** **3**a). Moreover, while water was used as the primary solvent here, other solvents which have a high polar component of surface tension are compatible with the process. Pure glycerol and ethylene glycol, with polar components of surface tensions of 30 mNm^−1^ and 19 mNm^−1^ respectively,^[^ ^32^ ^]^ are both compatible. Their lower surface tension led to a lower required surface energy for SA and so larger patterns for identical plasma treatment compared to deionized (DI) water (Figure S6, Supporting Information). This provides an additional vector for process optimization where inks can be tuned for either high‐resolution or high‐throughput processing.

The maximum area processing rate, which corresponds to the area treated to at least 1AU of surface energy per second, based on the characterization experiments was 1.1 mm^2^s^−1^ (Figure 3b), however stable self‐assembled structures could also be formed between treatments separated by up to 2 mm. By pattering an outline with partial infill, the processing time could be reduced substantially, as demonstrated for a circle with spiral infill in Figure 3c–e which took 25 s to process, as opposed to 286 s for the direct approach. The circle has a thicker center than edge due to the drying conditions and the formulation of the ink, but does not show any artefacts of the partial plasma treatment of the surface (Figures S7,S8, Supporting Information). Therefore, processing times can be optimized by using primarily the parameter set for high‐speed processing, including for large areas with infill as necessary, while switching in situ to the high‐resolution parameter set as required. This approach is enabled by the functionality of the apparatus to control the plasma discharge with low latency. For this approach, there is an upper limit on the pattern size as a result of hydrophobic recovery of the plasma treated regions, a well‐documented phenomena whereby the surface energy of the treated PDMS decreases with time.^[^ ^33^ ^]^ Here, due to the distribution of surface energy that results from the localized plasma source whereby the edges of the pattern receive lower degrees of functionalization compared to the center, the patterned lines shrink as the delay between plasma treatment and SA is increased. The magnitude of this effect is therefore dependent on the exact parameters and pattern but was characterized for straight lines of various widths (Figure S9, Supporting Information). Using the optimized high‐speed parameters of 4.5 kV and 6.0 kHz, at 8 mms^−1^ lines become discontinuous 4 minutes after patterning, while at 6.5 mms^−1^ they shrink to 50 µm after approximately 20 minutes. Therefore, for large patterns with long processing times it is possible to compensate for the hydrophobic recovery of the substrate by adjusting the speed of the patterning throughout. Using this approach, with an initial velocity of 6.5 mms^−1^ and linearly accelerating throughout the patterning up to 8.0 mms^−1^ over 17 minutes (leaving 3 minutes for the SA), gives a maximum pattern length of 7.395 m for this particular apparatus design. Using the infill approach from Figure 3c, this could create a circle of diameter 134 mm. As this is a function of the speed/processing rate, the maximum size could be increased by other apparatus configurations such as using a larger nozzle or multi‐nozzle arrays.^[^ ^34^ ^]^

The thickness of the deposited material is a function of the specific pattern geometry, the ink loading used, and the amount of ink introduced. Therefore, each of these aspects needs to be tuned based on the specifications of the desired device to give the required physical properties such as resistance or magnetization. Furthermore, the ink composition, in addition to the drying conditions, also effects the distribution of particles post‐drying. Here inks were designed to produce uniform distributions for narrow lines (<1 mm) through the inclusion of glycerol to suppress the coffee ring effect.^[^ ^35^ ^]^

**Figure 4:**
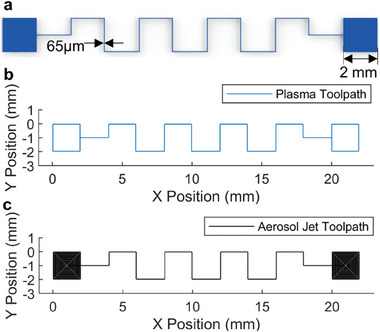
A comparison between self‐assembly via surface energy patterning and aerosol jet printing. a) A representative design. b) The toolpath self‐assembly via surface energy patterning would use, length 47.21mm. c) The toolpath aerosol jet printing would use, length 158.47mm.

To highlight the advantages of this process, it was compared directly to similar recent protocols for the manufacture of PEDOT:PSS structures on a polymeric substrate from template‐based and selective processing techniques; namely transfer printing^[^ ^36^ ^]^ and aerosol jet printing respectively.^[^ ^37^, ^38^ ^]^ This was evaluated by comparing the time taken to produce the representative structure shown in **Figure** **4**a, which is analogous to typical designs used in electronic devices, featuring two connection pads and a narrow interconnect. The toolpath to manufacture this pattern using SA via surface energy programming is shown in Figure 4b, with a total toolpath length of 47.21 mm. Using the optimized parameters results in a processing time of 5.9 s. The subsequent fluid SA then taking approximately 30 s, for a total production time of 35.9 s. Figure 4c shows the toolpath required for aerosol jet printing, the notable difference being the additional length required for the connection pads infill resulting in a total toolpath length of 158.47 mm. To achieve a printing resolution of 65 µm, typically a nozzle travel speed of 1–2 mms^−1^ is used.^[^ ^36^, ^37^ ^]^ At 2 mms^−1^ this results in a processing time of 79.2 s, ≈2.2 times longer than using SA via surface energy programming. The comparison to transfer printing is more complex due to the different characteristics of the approaches, so first solely the patterning steps in the procedure documented by Volkert et al. will be considered.^[^ ^36^ ^]^ In their approach, PEDOT:PSS films are selectively etched by reactive ions for 4 minutes, with the regions of the film intended to be transferred to the substrate protected by a metal mask. Subsequently, the patterned film was then transfer printed onto the substrate via a hydrogen bonding mechanism, taking 2 minutes. This totals 6 minutes of processing time, longer than either selective process. However, this time is independent of the size or number of the patterns produced, so it can be expected for the efficiency of transfer printing to grow as the production volume scales provided the design remains constant. If designs are changed, for example during prototyping or for personalized devices, then new masks must be fabricated, introducing substantial additional cost and lead time. These processing times should also be considered in the context of the unique additional steps of each process (steps in‐common such as ink preparation or solvent evaporation are not considered). For SA via surface energy programming, that is a 15‐minute helium purge prior to the first patterning. For aerosol jet printing this would include loading of the ink, stabilizing jetting parameters, and, for printing of high surface tension inks onto low surface energy substrates, such as PEDOT:PSS on PDMS, global plasma treatment of the substrate. Moreover, substantial cleaning or replacement of the aerosol pathway is required after printing or for material changes to prevent clogging and contamination. For transfer printing, there are additional steps associated with preparing the substrate for the PEDOT:PSS film, including its cleaning and UV Ozone treatment. Furthermore, a mask must be manufactured, the impact of which is dependent on the method used, but will increase the required equipment, cost, and lead time. For the feature sizes of this pattern, a laser micromachining method would be appropriate which incurs the need for additional apparatus, adds substantial processing time, and requires the use of strong acids for cleaning.^[^ ^39^ ^]^ Outside of this specific analysis, **Table** **1** details further general comparison of this SA approach versus aerosol jet printing, ink jet printing and template‐based methods. Based on these comparisons, it can be concluded that SA via surface energy programming offers substantial processing benefits for some applications versus the current state‐of‐of‐the‐art methods for low‐volume manufacturing.

**Table 1:** Comparison of template‐based printing, ink jet printing, aerosol jet printing, and self‐assembly via surface energy programming.

Process	Maximum resolution	Material requirements	Equipment cost	Advantages	Disadvantages	Refs.
Template methods	<100nm	Several materials can be processed if they can be appropriately formatted. (e.g., into ink, or precursor for vapour deposition)	Moderate to very high, method dependent	‐Very high resolution possible ‐Scalable to mass manufacture	‐Requires Template ‐Inefficient for low volume manufacturing ‐ Can have high infrastructure requirements (e.g clean room) ‐ Can require harmful chemicals (e.g photoresists, halogenated solvents, strong acids)	[36, 40, 41, 42]
Aerosol jet printing	6µm	1‐1000 mPa·s	High	‐High Resolution ‐Can print on non‐ planar substrates ‐ Design flexibility ‐ Large ink rheology range ‐ Many substrate materials	‐ Slow ‐ Regular cleaning required ‐ Nozzle clogging ‐ Requires several ml of ink for atomizers	[14, 37, 40, 43]
Ink jet printing	30 µm	1<Z<10, particle size < 2% of nozzle diameter	Low to high, spec. dependent	‐Multi‐material ‐High throughput by nozzle arrays ‐Design Flexibility ‐ Many substrate materials ‐ Low waste	‐ clogging ‐ limited ink rheology (Z number) ‐ lower resolution	[13, 17, 20, 40, 42, 44]
Self‐Assembly via Surface Energy Programming	12.5µm	Polar solvents Viscosity: <1 mPa·s (water) to 1300 mPa·s (Glycerol at 21 °C)	Low	‐ High resolution ‐ Large ink rheology range ‐Design flexibility ‐ High throughput for large areas ‐Low maintenance ‐ Easy material switching ‐ Low waste	‐Only one demonstrated substrate so far ‐Deposition thickness complex to control ‐Pattern size limited by hydrophobic recovery ‐ Limited to planar substrates	This work

## 3 Self‐Assembly of Functional Devices

**Figure 5:**
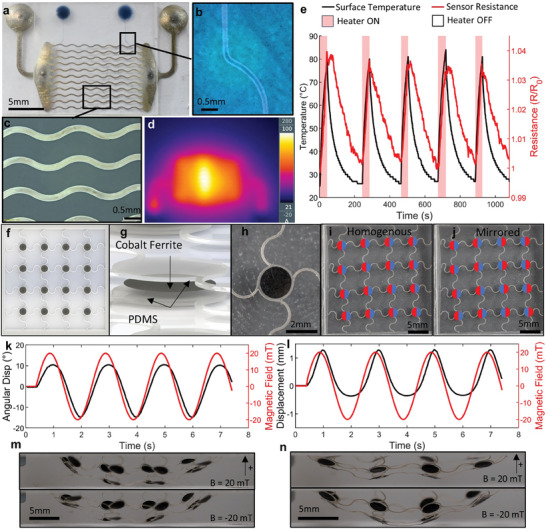
Functional demonstrators produced by surface energy programming. a) Self‐assembled silver heater with PEDOT:PSS temperature sensor. b) Magnified image of PEDOT:PSS track. c) Magnified image of silver heating elements. d) Thermal image of heater operating at 100 °C. e) Heater behavior and sensor feedback for 5 cycles to 80 °C surface temperature. f) Design of 16 element magneto‐mechanical film. g) Design of cobalt ferrite element. h) Image of cobalt ferrite element and connecting PDMS springs. i) Magnetization profile of homogenous film. j) Magnetization profile of mirrored film. k) Response of homogenous film to sinusoidal applied magnetic field. l) Response of mirrored film to sinusoidal applied magnetic field. m) Homogenous film at 20 and ‐20mT. n) Mirrored film at 20 and ‐20mT.

To showcase the potential impact of SA via surface energy programming, a flexible electronic large‐area silver heater with micro‐scale features and integrated PEDOT:PSS temperature sensor was manufactured (**Figure** **5**a–c, Figure S10, Video S2, Supporting Information). This was selected in reflection of the materials’ widespread use across flexible electronics applications and to demonstrate the ability of the process to satisfy the differing requirements of sensing and heating components without hardware modification. Despite the combined width of the heating elements being 30% greater than that of the supply lines and with a path length 0.5 mm (3%) shorter, their resistance is 133% greater at room temperature. This is due to the characteristics of the SA, whereby wider lines retain greater amounts of ink per unit width. Furthermore, the perimeter patterning approach offered a substantial time saving versus direct patterning. These two considerations contrast with typical direct‐write processes. These would pattern the design in its entirety, typically using a rastering approach at the minimum resolution required (200 µm for the heating elements), and multiple passes would be required to create the thickness differential achieved here. Both factors would extend the processing time substantially. The total resistance of the device was 20 Ω, with the heater elements accounting for 14 Ω. Applying between 0–5 V (corresponding to 0–1.25 W) resulted in localized heating in excess of 100 °C (Figure 5d). The surface energy programming required 56 s to complete, highlighting the unprecedented processing speed of the technique compared to other direct‐write processes. The heating element was complemented by a PEDOT:PSS temperature sensor (Figure 5a,b; Figure S10, Supporting Information). The fabrication of the sensor was completed immediately after the heater without hardware modification. This is also in contrast to typical direct‐write machines that would require material change procedure (cleaning, cartridge switching, etc.) or complex multi‐tool systems which are expensive and scale poorly to large materials libraries. The electro‐thermal behavior of both the sensor and the heater can be seen in Figure 5e, where it was cycled to 80 °C through an applied voltage of 5 V, with the sensor showing a temperature coefficient of resistance of approximately 0.075% °C^−1^. The sensor response shows a short lag following surface temperature decrease due to the encapsulation layer insulating the sensor, and the residual heating from the heating elements. This lag could be reduced by reducing the thickness of the insulating PDMS layer. This device demonstrates the capability of the approach to realize thin‐film electronics on stretchable substrates comparable to state‐of‐the‐art devices,^[^ ^45^, ^46^ ^]^ but with substantial processing benefits.

To further exhibit the technique, a programmable magneto‐mechanical film was fabricated capable of linear and angular displacement driven by torque generated from an externally applied magnetic field. In addition to the already demonstrated processing benefits, this approach addresses the long processing times required to pattern sufficient volume of magnetic material necessary for many applications suffered by bottom‐up fabrication approaches of micro‐magnetic devices.^[^ ^47^ ^]^ Furthermore, to the best of the authors’ knowledge, this is the first technique capable of maskless patterning of monolithic hard magnetic structures onto polymeric substrates.^[^ ^48^ ^]^ The design features 16 circular monolithic ferromagnetic CoFe_2_O_4_ elements across a laser cut PDMS film to produce a stretchable and magnetically active film (Figure 5f–j; Figure S10c, Supporting Information). CoFe_2_O_4_ nanoparticles were chosen owing to their hard magnetic properties with high intrinsic magnetocrystalline anisotropy (K, ∼ 2 × 10^5^ Jm^−3^), ease of synthesis, and dispersibility in aqueous solutions (see methods for synthesis).^[^ ^49^, ^50^, ^51^ ^]^ The prepared nanoparticles displayed a magnetization of 40.1 Am^2^kg^−1^ and coercivity of 342 kAm^−1^, recorded using vibrating sample magnetometry (superconducting quantum interference measurement device, Figure S11, Supporting Information). This contrasts with the super‐paramagnetic particles deposited via aerosol jet printing that has been demonstrated previously.^[^ ^52^ ^]^ The hard magnetic properties are key in applications which require magnetic torque, including the demonstrator device presented here which would not function with soft magnetic particles (including super‐paramagnetic particles). The CoFe_2_O_4_ elements were self‐assembled using a circular perimeter toolpath (Figure S10c, Supporting Information), with a total processing time of 40 s. Subsequently, the ink was dried, encapsulated with PDMS, and laser cut (see Methods). Two magnetization profiles for the film are demonstrated, termed “homogenous” (Figure 5i) and “mirrored” (Figure 5j). When an external magnetic field is applied, a torque is generated according to the difference in the orientation of the applied field and the magnetization of the CoFe_2_O_4_ elements, causing actuation which can be seen in Figure 5k,l for the homogenous and mirrored films respectively (Video S3, Supporting Information). The elements of the homogenous film move with identical patterns (with different initial orientations due to gravity). The selected element to be analyzed produced angular displacement between −15.3° and 10.5°. In the mirrored film, the opposing rotation of the elements produced an asymmetric linear deflection of the film between −0.35 mm and 1.3 mm between ±20 mT. These devices demonstrate the ability of this approach to realize actuatable structures featuring precise programable magneto‐mechanical behaviour rapidly and economically with architectures analogous to those deployed across magnetic robotics.^[^ ^53^, ^54^, ^55^, ^56^ ^]^

## 4 Conclusions

Surface energy programming presents a unique fabrication paradigm, combining the benefits of template‐based approaches (high throughput, material compatibility, economic scalability) with those of selective processing methods (flexibility, design freedom), whilst aligning with global initiatives towards environmentally friendly manufacturing. These benefits are manifest by leveraging fine digital control over the spatial distribution of plasma discharge, surface energy increase, and subsequent material arrangement from the micro‐ to meso‐scale. The demonstration of three frequently used functional materials already positions the technology for impact across a diverse range of fields, and the growth of a material library will be accelerated by the rapid characterization technique detailed. Consequently, this approach has the potential to disrupt current manufacturing models, firstly by enabling novel designs, materials, architectures, and behaviors, and secondly by offering a production‐volume independent and economically scalable manufacturing route to support their development and exploitation.

## 5 Experimental Section

### Substrate Preparation

Firstly, glass slides (76 × 52 × 1 mm) were cleaned using detergent wipes and DI water before drying with compressed air. Subsequently they were coated with high temperature release agent (Easy Release 200, Smooth On Inc.) and left to dry in air for 5 minutes. While drying, liquid sylgard 184 (PDMS) was prepared according to supplier guidance and degassed using a planetary mixer degasser (Thinky ARE‐250). Then, a PDMS coating was applied via spin coating onto the prepared glass slides. Three spin profiles were used depending on the desired film thickness; 2000 RPM for 60 s to achieve ≈30 µm, 1000 RPM for 60 s to achieve ≈60 µm, and 500 RPM for 30 s followed by a 5 hour planarization by gravity to give a 200 µm film.^[^ ^57^ ^]^ Once coated, slides were cured at 100 °C for one hour, per supplier guidance. PDMS was removed from the edges of the glass slide post‐cure to reveal the bear glass edge, which could act as a datum for processing. Prepared substrates were stored in a sealed container and care was taken to prevent dust contamination.

### CoFe_2_O_4_ Synthesis

CoFe_2_O_4_ nanoparticles were synthesized using a co‐precipitation method.^[^ ^49^ ^]^ A solution containing 0.1 M Iron(III) chloride hexahydrate(FeCl_3_.6H_2_0) and 0.05 M Cobalt(II) chloride hexahydrate (CoCl_2_.6H_2_0) was prepared in 300 ml DI water. Separately, 150 ml of 0.8 M sodium hydroxide (NaOH) solution was heated to 80 °C on a hot plate (UC150, Stuart) whilst being stirred, before the mixed salt solution was added dropwise. The formation of the ferrite nanoparticles was evident as the solution turns black. Once all the salt solution was added, stirring was continued for 2 hour at 80 °C before cooling to room temperature. Subsequently, to remove any biproducts or unreacted residues, the solution was washed until clean (typically 5 cycles) with DI water and collected by centrifugation. The final powder was dried in an oven at 60 °C for 1 hour, crushed to fine powders in an agate mortar and stored in an airtight container. To functionalize the nanoparticles for stable dispersion in water, equal weight of CoFe_2_O_4_ nanoparticles and citric acid (HOC(CH_2_CO_2_H)_2_) was taken. The citric acid was dissolved in 200 ml of water while stirring on a hotplate at 70 °C. The ferrite nanoparticles were added to the solution and stirred for 1 hour at 70 °C before cooling to room temperature. The solution was washed with DI water until clean (typically 5 cycles) and finally collected by centrifugation (MSE Harrier R, Medical Science Equipment). The powder was dried at 60 °C for 1 hour. The functionalized powder was redispersed into an ink (see Ink Preparation in methods).

### Ink Preparation

Three functional material inks were prepared; CoFe_2_O_4_, silver, and PEDOT:PSS. The silver and PEDOT:PSS inks used commercially available precursors, JS‐A426 (Novacentrix) and PH1000 (Ossilla) respectively. The CoFe_2_O_4_ was synthesized as detailed. Each functional precursor was mixed with DI water and glycerol according to ratios shown in Supplementary information Table S2 (Supporting Information). For the initial dispersion of CoFe_2_O_4_, the ink was sonicated using a probe sonicator (Q700, Qsonica). This was not required for other inks as they were provided in already stable suspension. Prior to use, all inks were shaken vigorously and then sonicated for 15 minutes in a water bath (FB15049, Fisherbrand).

### Plasma Jet Description

The plasma jet consists of a glass micro nozzle (TIP5TW1, World Precision Instruments), with a copper wire suspended approximately 1 cm from the aperture of the nozzle. The nozzle was set with epoxy into a custom adapter to enable interfacing with a pressurized helium supply, the flow from which was regulated by a digital gas mass flow controller (MFC, MC‐50SCCM, Alicat). The MFC was controlled and monitored via 0–5 V analogue signals from a MyRIO (National Instruments) micro controller running LabVIEW. The MyRIO also controlled an arbitrary function generator, used to generate the waveform to be amplified to generate the plasma. The waveform was amplified by 2000 times using a linear voltage amplifier (Trek 20/20‐HS), the output from which was connected to the copper wire within the nozzle. The nozzle sub‐assembly was mounted on a 3‐axis motion platform (MKS Newport) which communicated with the MyRIO via digital signaling to trigger and set the plasma parameters synchronized with the motion of the nozzle.

### Self‐Assembly Process

Prior to plasma treatment, helium was purged through the nozzle for at least 15 minutes to remove air contaminating the system. This was confirmed the be sufficient as the different ratio of specific heats between helium and air means the choked flow rate of the system can be used to monitor the helium content. Subsequently, a prepared substrate was placed onto the bed against a datum. Following this, the desired plasma treatment was initiated using the motion controller interface. Several process parameters were kept constant across all experiments, these were offset distance (0.5 mm), gauge pressure (199 kPa), waveform shape (sinusoidal), and waveform offset (0 V). Once completed, the treated sample was removed from the machine, and ink was introduced using a pipet manually at the approximated position of the plasma treatment. Doing so quickly minimized hydrophobic recovery of the PDMS substrate. The volume of ink introduced was controlled depending on the pattern and desired amount of final material. For wide traces (>0.7 mm), inks would immediately be attracted along the region of plasma treatment autonomously, or with a small amount of assistance, for example, by tilting the substrate (Video S1). Conversely, narrower traces would require the droplet to be manipulated through motion of the substrate such that the entire pattern was traversed. This could be achieved by a variety of methods such as shaking, tilting, or tapping the substrate. The threshold where energy input was required for ink assembly was a function of the specific pattern and ink rheology, for example highly viscous inks would require greater energy input to encourage ink spreading. This could also be integrated into an automated system. Any excess ink was removed from the substrate by pipet, or by manoeuvring off the edge of the substrate. Once the ink had achieved complete patterning it was placed on a hot plate heated at 130 °C to evaporate the solvent. Drying times varied between 30 seconds to 30 minutes depending on the volume of ink. Once dry, samples were ready to use or undergo further processing, for example encapsulation or sintering.

### Laser Cutting

Laser cutting was conducted using a UV laser system (Meta‐C UV 3 W, Lotus Lasers). To cut all samples a pulse duration of 0.05 µs at 40 kHz and scan speed of 100 mms^−1^ was used. For a 60 µm film, 10 passes were required, producing a cut width of 20 µm. At 200 µm, 30 passes were required to cut the films completely.

### Estimation of Nozzle Etch Rate

Helium was supplied at 199 kPa gauge pressure and allowed to flow unrestricted through the nozzle for 30 minutes. Then, the average flow rate was measured over 3 minutes to determine the initial nozzle diameter, see the Supporting Information S1 for how this relationship was derived. Then, the plasma discharge was turned on for 3 minutes using 4.5 kV at 6 kHz and typical processing conditions. Afterwards, the flow was allowed to stabilize for 1 minute, and then the average flow rate was measured over 3 minutes. The new nozzle size was then computed using the relationship found in the Supporting information S1. This was repeated 3 times and the average etch rate was reported.

### Characterization of Hydrophobic Recovery

Straight lines were plasma treated using 4.5 kV at 6 kHz using speeds of 6.5 mms^−1^ and 8 mms^−1^. The self‐assembly was then done at various delays after plasma treatment based on the previously found recovery time. Each was repeated 3 times.

### Fabrication of the Heater with Temperature Sensor

To fabricate the heater, first a substrate with 200 µm PDMS film was positioned against the datums on the bed of the machine. This film thickness was selected to make the device easy to handle once released from the glass slide. The plasma treatment was then executed at 8 mms^−1^ for the perimeter, and 6 mms^−1^ for the serpentine heating elements, using 4.5 kV and 6 kHz. The toolpath for which can be found in Figure S10a (Supporting Information). Once treated, silver ink was introduced in the approximate position of the plasma treatment and manipulated around the treated region (Video S2, Supporting Information). Excess ink was removed by pipet, and the substate was heated initially on a hotplate at 130 °C for 30 minutes to evaporate the ink, followed by a 15‐minute sintering at 200 °C in an oven to improve conductivity. The film was then laser cut and removed from the glass slide by peeling. Care was taken during the peeling to minimize the applied strain which may have affected the resistance. The fabrication of the sensor was largely the same as the silver heater except a different toolpath was used on a 60 µm film instead (Figure S10b, Supporting Information), and no post‐drying sintering was carried out. Once both the sensor and heater had been removed from their glass slide, the sensor film was placed on top of the heater. This was followed by another 200 µm film with the same dimensions as the heater but featuring holes cut to access the interface pads. This was selected to match the thickness of the heater substrate such that the heater and sensor elements were close to the neutral axis when bending, reducing strain. Using thinner layers would suppress the observed lag between the sensor and measured surface temperature. Wires were then attached to the interface pads using silver paint (RS Pro Silver Conductive, RS Components) for both the sensor and heater.

### Heater and Sensor Characterization

The heater was connected to a variable power supply capable of delivering up to 3 A and 30 V. The voltage limit was set to 5 V. The PEDOT:PSS sensor was connected to a digital multi‐meter to record resistance values (2450 Sourcemeter, Keithley Instruments). An external thermal camera (Testo 865, Testo, emissivity set to 0.95) was used to monitor the surface temperature of the sensors’ encapsulation. The heater power supply was cycled between 0 V and 5 V manually to change the temperature between ≈25–80 °C, while the surface temperature and resistance were monitored continuously.

### Fabrication of Magneto‐Mechanical Films

To fabricate the magneto‐mechanical films, first a substrate with 30 µm PDMS film was positioned against the datums on the bed of the machine. The plasma treatment was then executed at 8 mms^−1^ using 4.5 kV at 6 kHz using to produce the 4 by 4 array of 2 mm circular depositions. The CoFe_2_O_4_ ink was then introduced and manipulated around the substrate manually until the entire pattern had been traversed. Excess was removed by manipulating the droplet off the edge of the substrate. Subsequently, the substrate was heated at 130 °C to dry the ink before cooling to room temperature. After cooling, another 30 µm layer of PDMS was spin coated to encapsulate the CoFe_2_O_4_. The film was then laser cut and removed from the glass slide by peeling. Finally, it was placed onto a supporting jig to position the elements for magnetization, which was carried out under a uniaxial impulse field of 2.5 T (ASC IM‐10‐30, ASC Scientific). The films were then ready to use.

### Magneto‐Mechanical Film Characterization

To test the magnetic response of the magneto‐mechanical films, each was secured within an alignment frame and positioned within the workspace center of a 3D magnetic field generating system (MFG‐100, Magnebotix). A homogeneous magnetic field was subsequently supplied in the direction normal to the sample surface with an alternating sinusoidal oscillation profile of 20 mT amplitude and frequency of 0.5 Hz. The motion of each sample was concurrently imaged (D7500, Nikon) and the data processed to determine the motion profiles of selected sample nodes in Matlab.

### Vibrating Sample Magnetometry

To characterize the magnetic properties of the CoFe_2_O_4_ powder, a superconducting quantum interference measurement device vibrating sample magnetometer was used (SQUID VSM, Quantum Design). The hysteresis loops were measured at 300 K by cyclically applying a magnetic field up to ±1591.5 kAm^−1^

## Conflict of Interest

The authors declare no conflicts of interest.

## Author Contributions

L.J.T and R.A.H were responsible for the conceptualization. L.J.T was responsible for: the process development, characterization, and optimization; the model development and validation; all data analysis; and the design, manufacture, and characterization of the heater with temperature sensor. P.K was responsible for the synthesis and functionalization of CoFe_2_O_4_ nanoparticles. L.J.T and J.H.C were responsible for the design, manufacture, and characterization of the magneto‐magnetic film. The manuscript was drafted by L.J.T and R.A.H with input from all other authors. All authors were responsible for manuscript editing. R.A.H directed and supervised the research throughout.

## Supporting information



Supporting Information

Supplementary Video S1

Supplementary Video S2

Supplementary Video S3

## Data Availability

All source data from figures is available from the Research Data Leeds Repository.
