# Supplementary material for: High‐Resolution Self‐Assembly of Functional Materials and Microscale Devices via Selective Plasma Induced Surface Energy Programming
Source: Small. 2024 Dec 29;21(6):2408822. doi: 10.1002/smll.202408822 (PMC11817948; doi:10.1002/smll.202408822)
Supplement: Supplementary file 1 — Supporting Information [file SMLL-21-2408822-s002.docx]

**High-Resolution Self-Assembly of Functional Materials and Microscale devices via Selective Plasma Induced Surface Energy Programming**

*Authors: Luke J. Tinsley*, Prakash Karipoth, James H. Chandler, Silvia Taccola, Pietro Valdastri and Russell A. Harris**

L.J. Tinsley, P. Karipoth, S.Taccola, R.A.Harris

Future Manufacturing Processes Research Group, School of Mechanical Engineering, University of Leeds, LS2 9JT

Email: [L.J.Tinsley@leeds.ac.uk](mailto:L.J.Tinsley@leeds.ac.uk), [R.Harris@leeds.ac.uk](mailto:R.Harris@leeds.ac.uk)

J.H.Chandler, P.Valdastri

STORM Lab, School of Electrical Engineering, University of Leeds, LS2 9JT

**Contents**

S1 Analysis of gas flow

S2 Effect of plasma ignition on gas flow rate Supplementary Information

S3 Rapid Characterisation Method

S4 Toolpath Design

Table S1 Parameters used to validate line width model

Table S2 Ink Composition

Figure S1-S11

**S1 Analysis of gas flow**

As nozzles varied in size as an unavoidable consequence of their manufacture, and would have subsequently varied in etching during processing, it was necessary to develop a method of in-situ measurement to allow nozzles of certain sizing to be selected. This was done by monitoring the choked gas flow rate at a known input pressure. As the nozzle is only converging, that is, the cross-sectional area only decreases before reaching the atmosphere, the maximum speed of the flow is the local speed of sound at the nozzle outlet. There is a critical pressure ratio between the helium source and the atmosphere termination to achieve this, any pressure increase to the source beyond this will increase the pressure in the nozzle. Assuming the helium behaves as an ideal gas, the critical pressure ratio could be found using equation S1. All pressures and temperatures are absolute unless otherwise stated, and all units are SI unless state otherwise.

*(1)*

$$\frac{P_{d}}{P_{0}}={\frac{2}{\gamma+1}}^{(\frac{\gamma}{\gamma+1})}$$

Where $P_{d}$ is the downstream pressure, $P_{0}$ is the source pressure, and $\gamma$ is the ratio of specific heats (1.667 for helium). As the downstream pressure is atmospheric, using the value for a standard atmosphere (101.3 kPa) gives a critical source pressure of 207.55 kPa. If the source pressure is increased beyond this, there will be a resultant pressure difference between the inside of the nozzle and atmosphere. This was given by equation S2.

$$Pn=P_{0}({1+\frac{\gamma-1}{2})}^{\frac{\gamma}{\gamma-1}}-P_{d}$$

Where $Pn$ is the gauge pressure between the inside of the nozzle and the atmosphere. This pressure increase in the nozzle also leads to an increase in the density of the helium at the nozzle, so despite the velocity of the gas being limited to mach 1, the mass flow rate can still be increased. To find the density of helium in the nozzle, the temperature of the gas first had to be found using equation S3.

*(2)*

*(3)*

$$T=T_{0}{(1+\frac{\gamma-1}{2})}^{-1}$$

Where $T$ is the temperature in the nozzle, and $T_{0}$ is the initial temperature of the helium (294.15K). The gives a nozzle temperature of 220.6 K. The density could then be found using equation S4.

*(4)*

$$\rho=\frac{P_{n}+P_{d}}{RT}$$

Where $\rho$ is the density and $R$ is the gas constant (2077 Jkg^-1^K^-1^). The local speed of sound and cross-sectional area are also required to find the mass flow rate, given by equations S5 and S6 respectively.

*(5)*

*(6)*

$$v=\sqrt{\gamma RT}$$

$$A=\pi r^{2}$$

Where $v$ is the local speed of sound, A is the cross-sectional area of the nozzle, and r is the radius of the nozzle. The mass flow rate of the gas could then be found using equation S7.

*(7)*

$$\dot{m}=\rho vA$$

Now, for a given nozzle diameter and input pressure the maximum gas flow rate and corresponding pressure in the nozzle could be computed. This needed to be converted into SCCM, the equivalent volume flow rate of the gas at standard conditions, which is used by the digital mass flow controller. This was done using Equation S8.

*(8)*

$$\dot{V}=\dot{m}\rho_{std}\times60E6$$

Where $\dot{V}$ is the volumetric flow rate in SCCM, $\rho_{std}$is the density of helium at standard temperature and pressure, $60E6$is the conversion coefficient between m^3^s^-1^ and SCCM. By operating at higher input pressures the range of gas flow rate can be increased substatially, giving more flexibility to the process, but this also increases the pressure inside the nozzle which at a critical limit will break the nozzle. For all experiments, an absolute input pressure of 300 kPa was selected as a comprimise between these conflicting relationships. For the selected input pressure, the maximum gas flow rate for a range of nozzle sizes can be computed. By measuring the actual gas flow rate it is possible to calculate the size of the nozzle continously through the processing. This analysis assumes that the helium is behaving as an ideal gas, which is valid for all the conclusions above, but when the gas is ionised in the nozzle the conditions change substantially, violating the assumptions of ideal gas analysis. The actual conditions when the plasma jet is operating deviate from those found during this analysis.

**S2 Effect of plasma ignition on gas flow rate**

Due to the limits of analytic methods, we sought to measure the effect of the plasma ignition on the gas flow. To do so, the 300 kPa helium supply was connected to the nozzle with the flow unrestricted, to emulate the conditions of the analysis. Once the flow rate had stabilised, it was measured using the 0-5 V feedback signal from the MFC and recorded for 30 seconds at intervals of 20 ms. This process was repeated for all plasma parameters used in the systematic characterisation. The results can be seen in Figure S5. Note, the size of the nozzle at the time of these experiments was 6.35 µm, resulting in an unrestricted flow rate of 5.6 SCCM. As the voltage and frequency of the applied signal were increased, the gas flow rate decreased due to the ignition of plasma providing resistance to the overall flow. The sensitivity of this effect was highest at 4.5 kV, the highest voltage tested. While at 2 kV, the flow rate was independent of any increase in frequency.

**S3 Rapid Characterisation Method**

This section will describe the method transforming the data gathered using the experiment described above into a profile of how the treatment rate varies radially. Firstly, a series of stationary exposures were carried out for durations between 0.115s-19.240s. These were 0.115s, 0.190s, 0.340s, 0.640s, 1.240s, 2.440s, 4.840s, 9.640s and 19.240s. In principle any time periods could be used, but shorter durations than 0.115s were avoided to minimise the impact of the transient discharge which occurs during the initial ignition. Subsequently, the ink being tested would be self-assembled according to the methods detailed. The radius of each deposit was measured using a digital microscope (BX53M, Olympus). This radius corresponded to the distance from the nozzle which had received exactly enough surface energy increase to induce self-assembly for the given ink on a given substate. This was level of treatment was assigned value of 1 arbitrary unit (AU). Therefore, the treatment rate could be found by dividing the treatment level (always 1 AU) by the exposure time. Doing so for every stationary treatment gave a relationship for treatment rate vs radial distance from the nozzle. This is noted as:

*(9)*

$$\frac{dT}{dt}=f(D)$$

Where $T$ is surface energy increase in AU, t is time in seconds, and $f(D)$ is the empirically derived relationship between treatment rate and distance. To predict how wide a line will be for a given speed, the total treatment delivered needs to be calculated at position increments. The line width then corresponds to the position where the total treatment equals 1AU. The total treatment can be found by using equation S10.

*(10)*

$$T=\int_{t=-\infty}^{t=\infty} f(D)dt$$

Hence, a function which describes how the distance from the nozzle varies with time is required to find the total treatment. This function depends on the toolpath and speed. Firstly, this was done using a straight-line path at constant speed, and then applied to arbitrary paths. The distance between the nozzle and the position being evaluated can be found using equation S11:

*(11)*

$$D={(x^{2}+y^{2})}^{0.5}$$

Where $D$ is the distance to the nozzle, $x$ is the distance to the nozzle in the X axis and $y$ is the distance to the nozzle in the Y axis. For a straight-line path, one axis value is constant depending on the position being evaluated (Y chosen here), and the X position is a function of the speed and time. For a constant speed, this gives the position function:

*(12)*

$$D={(({vt)}^{2}+y^{2})}^{0.5}$$

Where $v$ is the x velocity and $t$ is the time. Now the position is expressed in terms of the velocity of the nozzle and an evaluation distance perpendicular to the travel of the nozzle, a specific treatment rate profile can be found for a given velocity and perpendicular distance. For example, considering a velocity of 2 mm/s and evaluating the treatment at 0.5 mm from the nozzle gives the following relationship:

*(13)*

$$D={(({2t)}^{2}+{0.5}^{2})}^{0.5}$$

Now the distance from the nozzle is a function of time. Substituting this function into equation S9 then gives the treatment rate as a function of time for the specific velocity and distance perpendicular distance being evaluated. This was interpolated using a piecewise cubic interpolating polynomial method in Matlab. The intergal of this function was then found using the trapezium rule. If this intergral is ≥ 1, then self assembly is expected to occur at this linewidth for this speed being evaluated. This process could then be repeated while incrementing $y$ to find where the integral equals 1, which corresponds to the expected linewidth for the given speed. This process can then be repeated for different velocities and for different voltage/frequency combinations. As this approach required thousands of calculations per parameter set, it was done computationally in Matlab, taking approximately 60 seconds to compute for each parameter set. In addition, it was also possible to compare the area processing speeds, by multiplying the speed by the line width, which could be used to find the optimal parameters for maximising treatment of large areas. As this process was a non-direct method of investigating the effect of the velocity, it was necessary to validate the method against real data gathered. To do this, the nine fixed duration exposures required for the line width prediction were carried out first, followed by 4 lines at different velocities, chosen based upon the expected line widths. These were all between 0.5 mm/s and 3 mm/s. The average width of the line was measured using a digital microscope, and then compared to the prediction. The validation process was done for 6 parameter combinations, shown in Table S1. The results can be seen in figure S2.

Across all the parameter combinations validated the prediction aligned well with the actual measured line width, with an average deviation from the prediction of 4.77%, standard deviation of 3.9%, and maximum error of 16.8% (n=23). This low error is expected as the predictions use empirical data which captures the conditions of the system, such as the exact atmospheric conditions, unlike in analytical modelling which typically use assumptions to simplify problems. Therefore, the only source of variation is from the process itself. This is reflected in the magnitude of error of this model. Based upon this, the prediction method outlined previously can be considered accurate and was valid to use instead of direct measurement of the line width.

Based on the principles validated in the 1D modelling, it was adapted to a computational 2D version which used the same CAD input as the apparatus to generate toolpaths. The substrate was discretised into in pixels typically of 20 µm by 20 µm. Each pixel has a stored value of surface energy in AU, initially at 0. The toolpath created from the CAD input is then discretised along its length into segments, typically of length 20 µm. This distance is then converted into a time, using the desired speed to be modelled. Based on this time and the input parameter data, the amount of surface energy increase that will be delivered over that segment is computed using the 1D method and added to the pixels representing the substrate according to their distance from the modelled position of the nozzle. The process was repeated for every segment of the toolpath, giving the predicted distribution of surface energy increase. To be valid, the segment length needs to be substantially smaller than the toolpath length. Approximately 3 s were required to produce the model output shown in Figure S1 using the typical element sizes detailed. Therefore, it was efficient to iterate designs based upon model outputs.

**S4 Toolpath Design**

Toolpaths were designed with three major considerations. Firstly, the speed and shape of the toolpath were designed to give the intended self-assembly pattern based upon the characterised spatial distribution of the plasma discharge. Secondly, where possible, paths were designed to be Eulerian to minimise the frequency with which the plasma was ignited and extinguished during which the discharge behaviour was transient, and to remove artifacts of system lag between signalling and execution of the intended behaviour. Finally, the acceleration required to drive the toolpath at a constant speed was ensured to be within the capabilities of the motion platform, as treatment distribution is highly dependent on speed.

**Table S1. Parameters used to validate line width model.**

| Validation | A | B | C | D | E | F |
| --- | --- | --- | --- | --- | --- | --- |
| Voltage(kV) | 2.5 | 3.0 | 3.5 | 4.0 | 4.5 | 4.5 |
| Frequency(kHz) | 12.0 | 10.5 | 8.5 | 7.25 | 6.0 | 2.0 |

**Table S2. Ink Composition**

| **Ink** | **DI Water (weight %)** | **Glycerol (weight %)** | **Solid Content(weight %)** |
| --- | --- | --- | --- |
| *Silver* | 73.0 | 15.0 | 12.0 |
| *Cobalt Ferrite* | 73.0. | 15.0. | 12.0 |
| *PEDOT:PSS* | 78.0 | 16.0 | 6.0 |


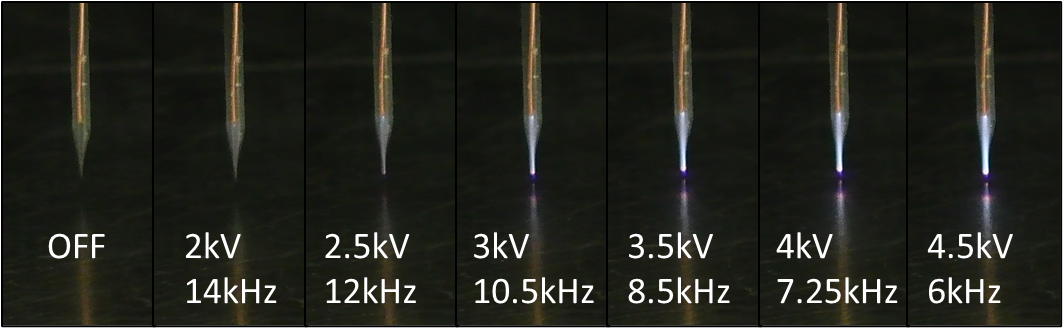
 **Figure S1 – Plasma Discharge**

Figure S1. Plasma discharge at different applied voltages and waveform frequencies between 0-4.5 kV and 6-14 kHz respectively.

**
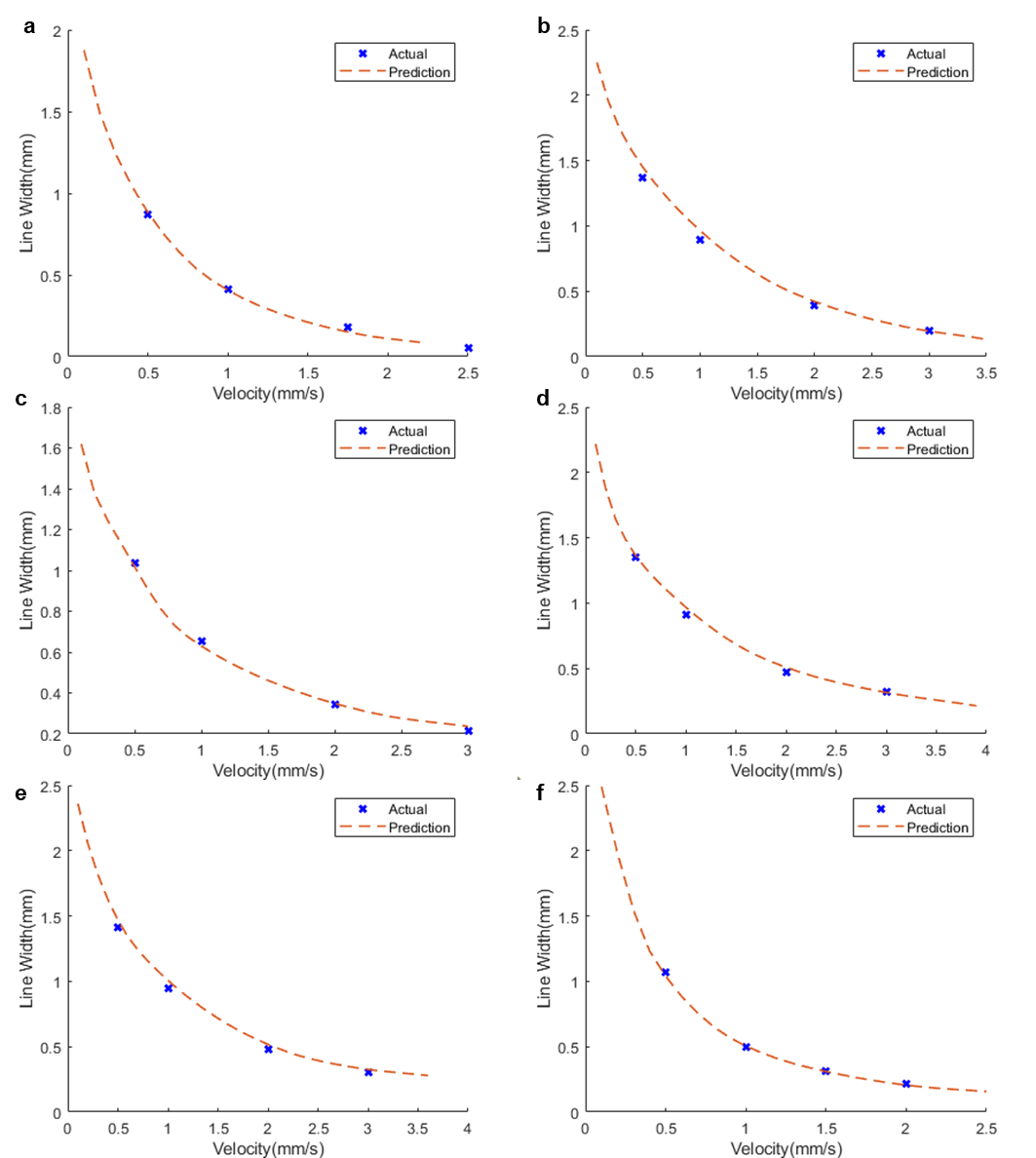
 Figure S2 – Model Validation**

Figure S2. Validation of model for transforming stationary discharge data into line width prediction for different parameter combinations. a) 2.5 kV @ 12.0 kHz b) 3.0 kV @ 10.5 kHz c) 3.5 kV @ 8.5 kHz d) 4.0 kV @7.25 kHz e) 4.5 kV @ 6.0k Hz f) 4.5kV @2.0 kHz

**Figure S3 – Line Width vs Velocity Data
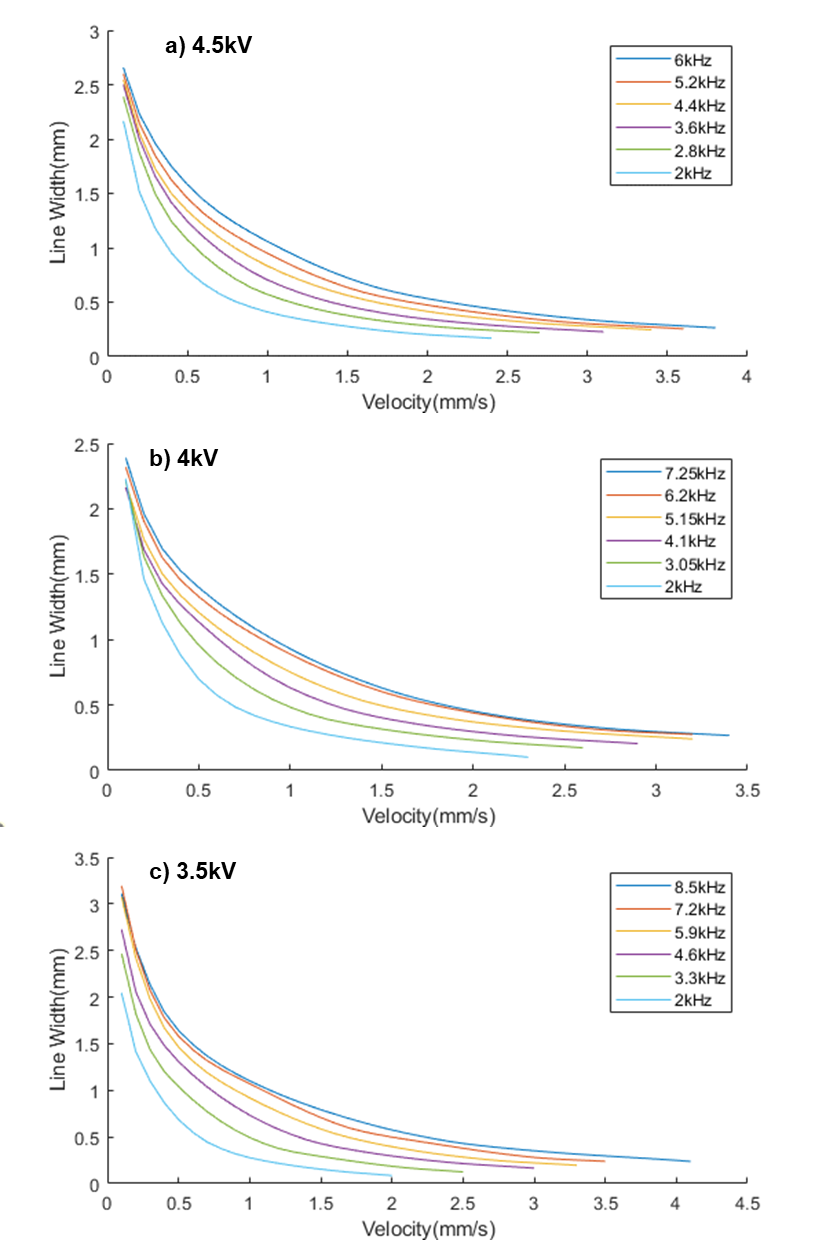
**

Figure S3. Line width vs velocity for all frequencies tested at voltages of a) 4.5 kV, b) 4.0 kV and c) 3.5 kV.

**Figure S4 – Further Line Width vs Velocity Data (Continued)**

**
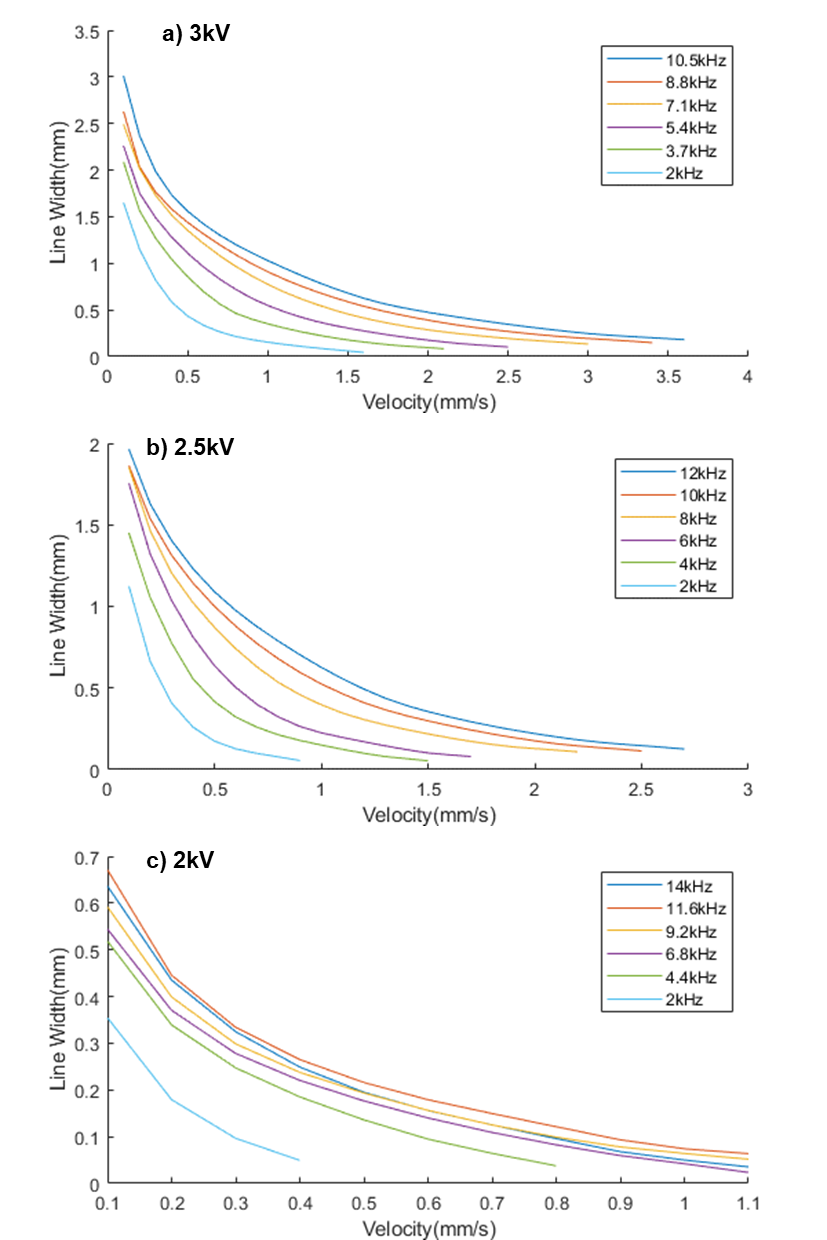
**

Figure S4. Line width vs velocity for all frequencies tested at voltages of a) 3.0 kV, b) 2.5 kV and c) 2.0 kV.


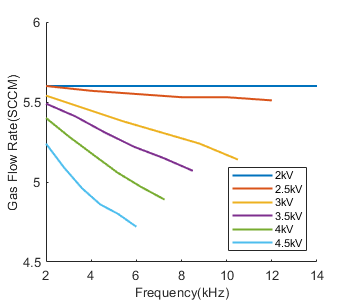
 Figure S5 – Effect of plasma on choked gas flow rate

Figure S5. The choked gas flow rate during plasma discharge for all tested parameter combinations.

**Figure S6. Effect of solvent surface tension on line width**

**
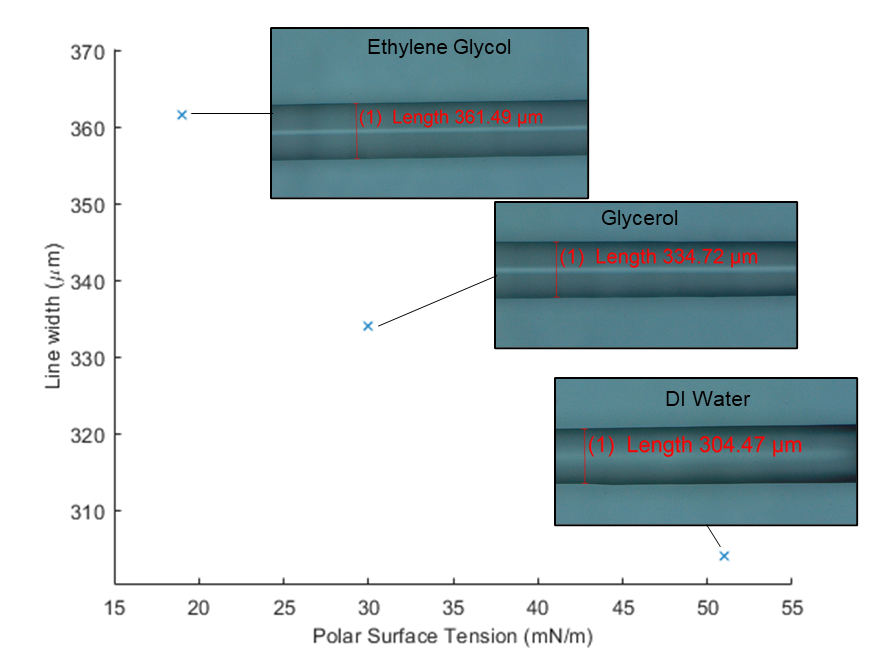
**

Figure S6. Line width for pure deionised water, glycerol, and ethylene glycol for line patterned using 4.5 kV 6.0 kHz at 4 mm/s.

**
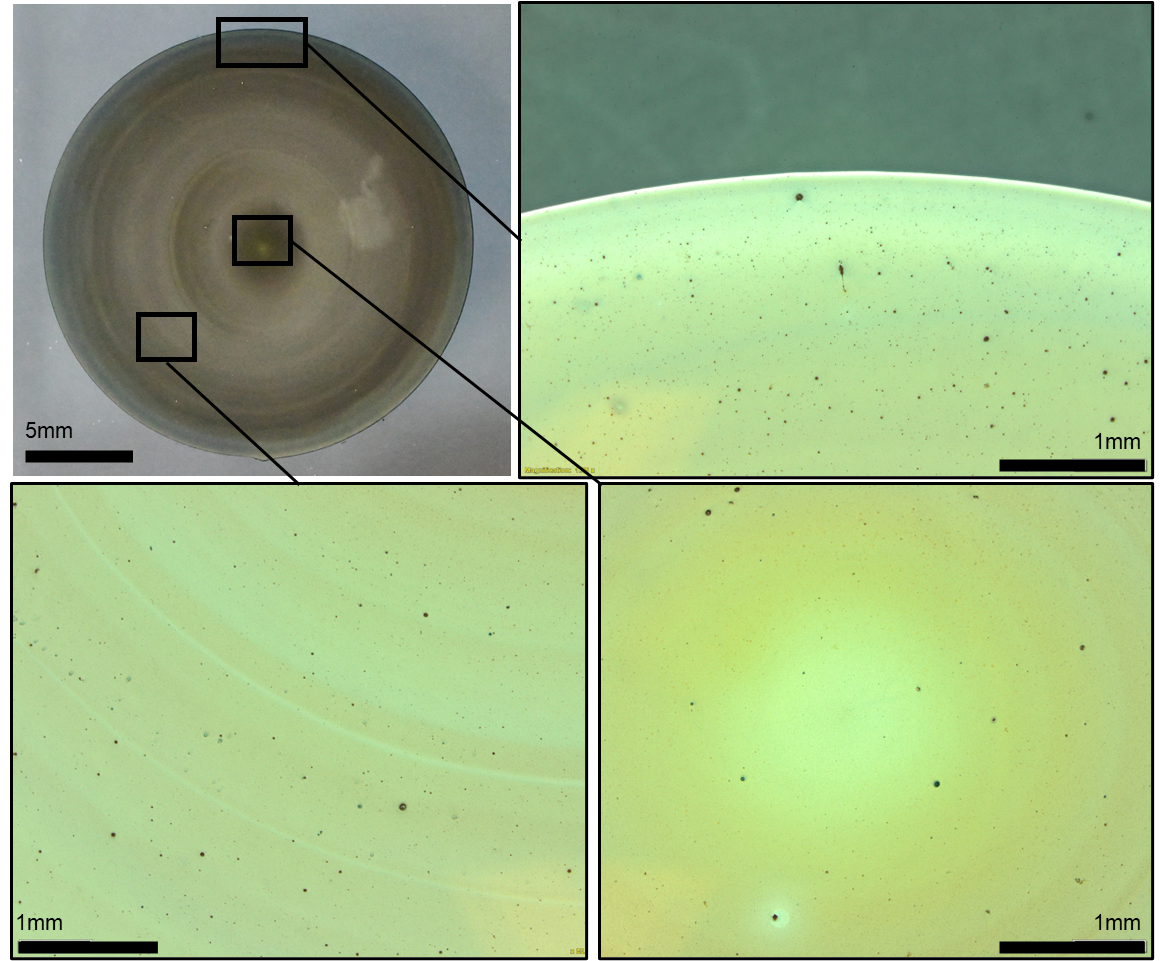
Figure S7. High Magnification Images of 20mm Silver Circle**

Figure S7. High magnification images of the silver circle fabricated through partial infill approach.

**
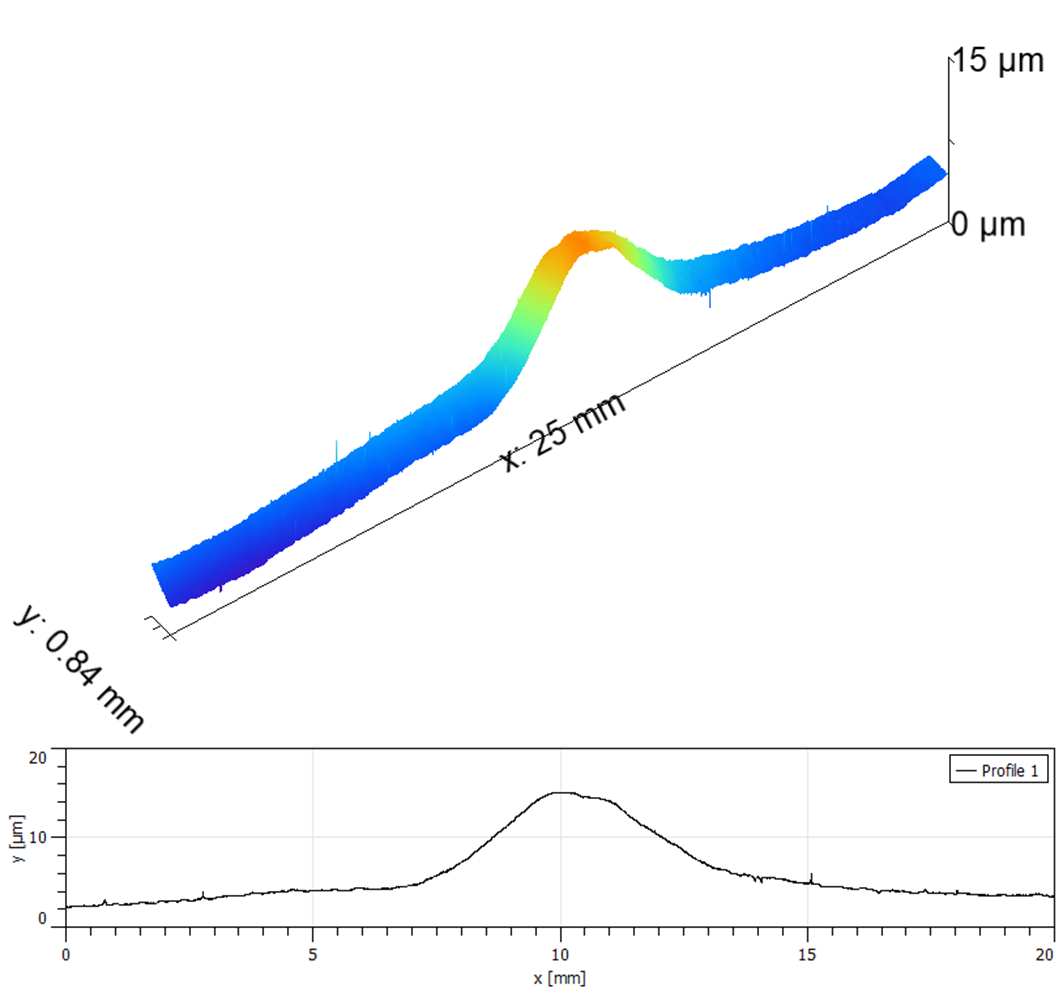
Figure S8. Height Profile of 20mm Silver Circle**

Figure S8. Profile of silver circle fabricated through partial infill approach. Data captured using white light interferometry.

**Figure S9. Hydrophobic Recovery of the Substrate**

**
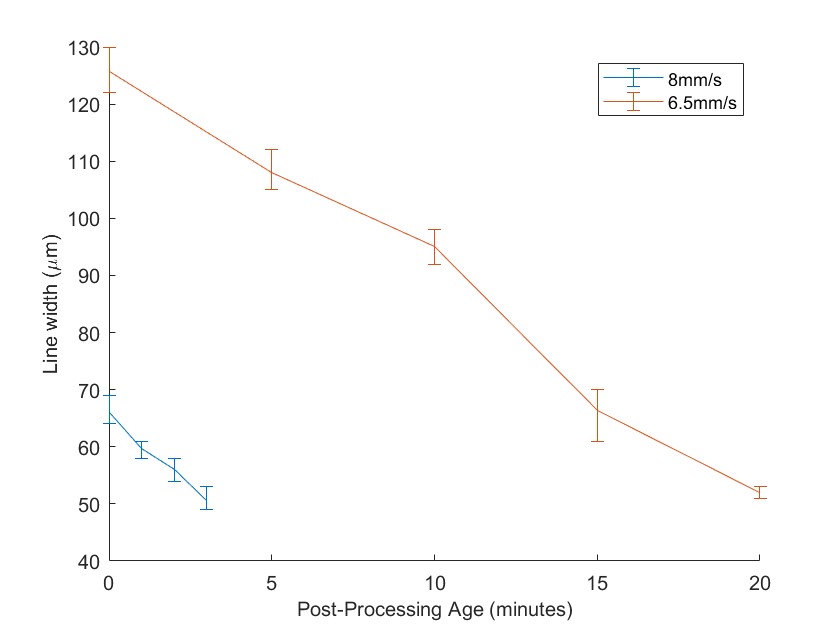
**

Figure S9. The magnitude of line shrinking as a result of hydrophobic recovery as substrates age post-treatment for two speeds using 4.5kV at 6kHz.

**Figure S10. Design and Toolpaths for Demonstrators
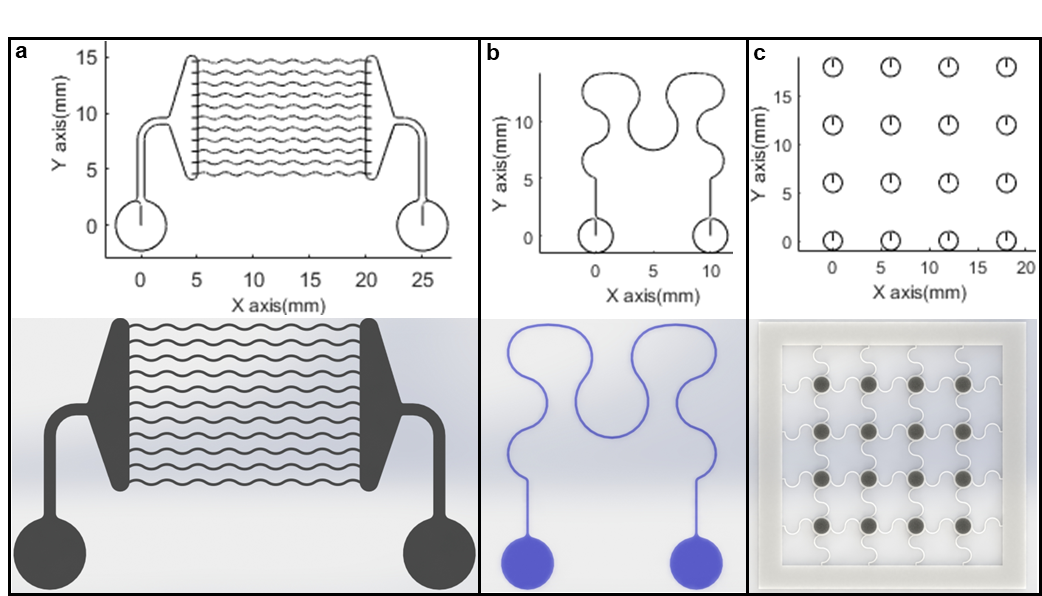
**

Figure S10. Toolpath and CAD data used to create functional demonstrators for a) the silver heater, b) the PEDOT:PSS temperature sensor, and c) the magnetomechanical film.

**Figure S11. Vibrating Sample Magnetometry of Cobalt Ferrite nanoparticles**


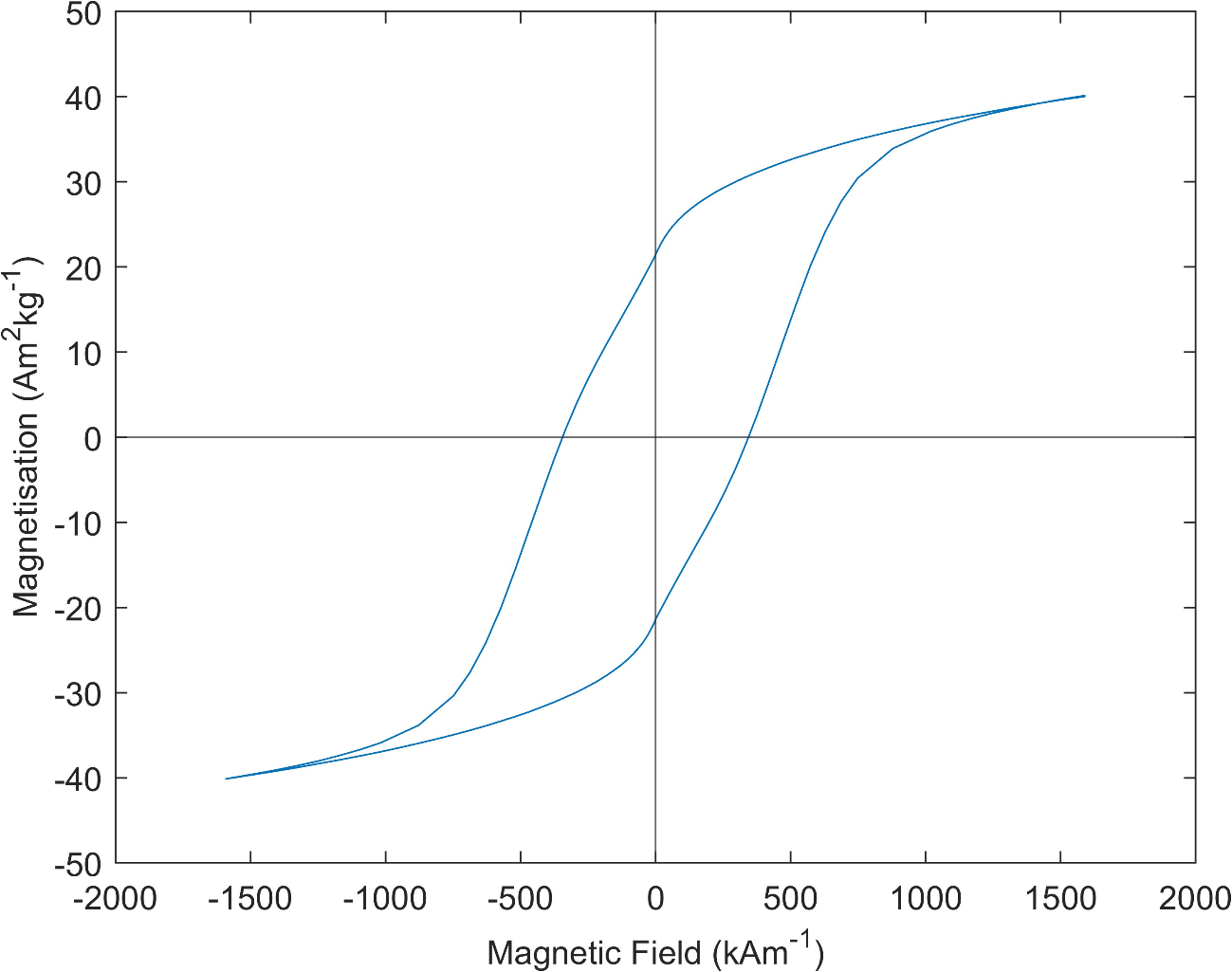


Figure S11. Superconducting quantum interference measurement device vibrating sample magnetometry results for the cobalt ferrite nanoparticles synthesised.
